# Imaging of polysaccharides in the tomato cell wall with Raman microspectroscopy

**DOI:** 10.1186/1746-4811-10-14

**Published:** 2014-05-29

**Authors:** Monika Chylińska, Monika Szymańska-Chargot, Artur Zdunek

**Affiliations:** 1 Institute of Agrophysics, Polish Academy of Sciences, Doswiadczalna 4, 20-290 Lublin, Poland

**Keywords:** Plant cell wall, Polysaccharides, Raman imaging, PCA, MCR

## Abstract

**Background:**

The primary cell wall of fruits and vegetables is a structure mainly composed of polysaccharides (pectins, hemicelluloses, cellulose). Polysaccharides are assembled into a network and linked together. It is thought that the percentage of components and of plant cell wall has an important influence on mechanical properties of fruits and vegetables.

**Results:**

In this study the Raman microspectroscopy technique was introduced to the visualization of the distribution of polysaccharides in cell wall of fruit. The methodology of the sample preparation, the measurement using Raman microscope and multivariate image analysis are discussed. Single band imaging (for preliminary analysis) and multivariate image analysis methods (principal component analysis and multivariate curve resolution) were used for the identification and localization of the components in the primary cell wall.

**Conclusions:**

Raman microspectroscopy supported by multivariate image analysis methods is useful in distinguishing cellulose and pectins in the cell wall in tomatoes. It presents how the localization of biopolymers was possible with minimally prepared samples.

## Background

The primary cell wall of fruits and vegetables is a heterogonous structure mainly composed of polysaccharides, including a wide variety of pectins and hemicelluloses as well as cellulose. Most plant cell wall components are important elements of human nourishment, known as dietary fiber [1]. The plant cell wall is a kind of cellular skeleton that controls cell shape and determines the relationship between turgor pressure and cell volume. The composition of plant cell walls is also important from the point of view of macroscopic mechanical properties and water transport [2-4].

The cell wall is a composite of cellulose, hemicellulose and pectin, with the addition of other, non-polysaccharide components like proteins, lipids, enzymes and aromatic compounds. Generally, the primary cell wall is composed of approximately 25% cellulose, 25% hemicellulose and 35% pectins, with up to 8% structural proteins (on a dry-weight basis). However, large deviations from these values may be found [5].

Cellulose is a highly stable and linear polymer containing between 1,000 and one million D-glucose residues linked by β-1,4 glycosidic bonds. Pectins and hemicelluloses are represented by wide variety of compounds [6,7]. Xyloglucan is the most common hemicellulose present in primary cell wall of higher plants [5,8]. Among pectins the main are homogalacturonan, rhamnogalacturonan I and rhamnogalacturonan II. According to the common plant cell wall model, the cellulose is organized in microfibrils, which are tethered and connected via hemicelluloses. The cellulose-hemicellulose network is embedded in a highly hydrated matrix composed of pectin polysaccharides [9]. It is suggested that the key role in the biomechanics of plant cell walls is played by the xyloglucan-cellulose network, which has loadbearing properties [10]. In addition, pectins are responsible for controlling cell wall porosity and inducing the binding of neighboring cells [11]. It is thought that the percentage of the components of the plant cell wall has an important influence on the mechanical properties of fruits and vegetables [12,13].

Up to now, many analytical [14,15] and microscopic (e.g., optical [16] and electron microscopy [17]) methods have been used for the evaluation of the plant cell wall’s structure and composition. So far, antibody probes have served as the most common and - probably - the only effective method to provide micro-scale insight into the fruit cell wall’s biochemical structure. The labelling of polysaccharides with antibodies was applied in several studies. Immunocytochemistry was used to depict the spatial deployment of pectins in potato cell walls [18] and in ripening tomatoes [19]. Also, the spatial distribution of hemicelluloses in fruit cell walls was investigated [20,21]. However, despite its many advantages, this method is very selective, quite expensive and time-consuming.

Raman imaging has been suggested as being a complementary or even alternative method to antibody labeling in studying the spatial distribution of fruit cell wall components [22]. In brief, Raman microscope is a connection of microscope and Raman spectroscope. Raman spectra are collected while scanning over a sample, providing the spatial and chemical labeling of various components within the sample simultaneously. In this way a map of spatial distribution of sample’s components can be obtained. However, the resolution is limited by the diffraction limit, which depends on the wavelength of the excitation light and the objective used. Typically it cannot be lower than 200 nm. This is insufficient to depict single polysaccharide in cell wall which have diameter of about few to several dozen nanometers however it could be used to visualize the spatial distribution at the micro-scale level. The Raman spectroscopy based on “Raman effect” was described for the first time in 1928 [23]. When a sample is illuminated by monochromatic laser light, mainly elastic scattering (“Rayleigh scattering”) of photons is observed . It means that the energy of incident radiations is equal to the energy of scattered light. However, a small amount of incident light (about one in a million photons) is scattered with different frequency. In case of such inelastic scattering, the energy of photons of scattered light can be lower or higher than the energy of incident light. In Raman spectrum lines corresponding to lower frequencies are presented as a plot of intensity versus frequency shift [24].

Raman microspectroscopy has been shown to be a useful method in the study of polymers and biopolymers [25]. One of the first applications of Raman imaging was in the evaluation of the distribution of chemical components in flex (*Linum usitatissimum* L.) stem tissue [25,26]. The potential of Raman microspectroscopy was frequently demonstrated on wood cell walls, like the distribution of lignin and cellulose in black spruce wood (*Picea mariana*) [27] and the lignin spatial content in the cell wall of poplar wood [22]. Furthermore, single carotenoid crystals were detected directly in carrot cells, without any compound extraction [28]. Vibrational spectroscopic imaging techniques - especially Raman microscopy - have seen numerous applications in the medical sciences (e.g., in cancer diagnosis) [29].

A literature review revealed a lack of an application of Raman imaging technique to the evaluation of the chemical composition and distribution of polysaccharides in the cell wall in fruits and vegetables. Taking into account the simplicity of Raman imaging and the relatively easier sample preparation (which does not require extensive chemical treatment prior to imaging), this method would benefit in more effective characterization of the composition of cell wall with particular emphasis on dynamic changes which occur during development and maturation of fruits and vegetables. However, at present an application of this method requires developing an approach to handle of a large set of data which combines both spatial and spectral information. Beside of traditional single band imaging, multivariate techniques could be used to visualize in a more complex manner the spatial distribution of cell wall constituents due to fact that each polysaccharide is represented in Raman spectra by more than one band. Therefore, the general aim of the present work is to depict the spatial distributions of the main cell wall compounds in tomato tissue using Raman imaging. The tomato fruit was chosen due to the fact that it has become a well-known model system in the study of fleshy fruits [30]. In order to evaluate the spatial and spectral data, three methods for the visualization of the distribution of polysaccharides in cell walls were used: single Raman band imaging for preliminary analysis, principal component analysis (PCA) and multivariate curve resolution (MCR).

## Results and discussion

### Assignment of Raman spectra to cell wall polysaccharides

Table 1 presents assignment of bands in the Raman bands characteristic for the most abundant plant cell wall polysaccharides based on the literature [31-33]. As a consequence of similar chemical compositions, many Raman bands originating from the vibrations of the same functional groups were overlapping for most of the polysaccharides [34]. For example, the band of ν(C = O)’s vibration at 1,742 cm^-1^ could be assigned to both hemicellulose (xyloglucan, glucomannan) and pectin [31,35]. Theoretically, this ester band allows us to distinguish between hemicellulose and cellulose. However, in practice, in plant cell wall samples this is almost impossible due to significant pectin interference. Nevertheless, it may be that there are some particular Raman shifts which are unique to each polysaccharide and which could be used for the identification of these compounds in the cell wall material. In the CH stretching region from 2,700 cm^-1^ to 3,060 cm^-1^ characteristic for carbohydrate, intense bands assigned to pectin, hemicellulose and cellulose were observed at 2,952, 2,930, 2,897 cm^-1^, respectively. The peaks at 1,121 cm^-1^ and 1,098 cm^-1^ are assigned to the symmetric and asymmetric stretching mode of COC in the glycosidic bond in cellulose, respectively, and it should be noted that these bands are highly sensitive to the orientation of microfibrils along the fiber. Bands centered at 1,378 and 971 cm^-1^ Raman shift might also indicate the presence of cellulose in the sample. Additionally, the band at 380 cm^-1^ related to the ring deformational modes is also characteristic for the cellulose. Moreover, the characteristic sharp band at 854 cm^-1^ can be considered to be a marker band for α-glycosidic bonds in pectin. In particular, this band is very sensitive to O-acetylation [36]. Therefore, it could take values from 850 to 862 cm^-1^ according to the increasing degree of pectin acetylation. On the average spectrum of the tomato cell wall, other bands denoting pectin at 817 and 478 cm^-1^ are also present.

**Table 1:** **Assignment of bands in the Raman spectra of cell wall polisaccharides based on the literature**[31-33]**: C – cellulose; P- pectins; H – hemicelluloses**

**Raman wavenumber [cm**^**-1**^**] (literature)**	**Assignment**	**Origin**
2952	ν(CH)	P
2930	ν(CH)	H
2897	ν(CH)	C
1742	ν(C = O) ester	P, H
1378	δ(HCC), δ(HCO), δ(HOC)	C
1256	δ(CH), δ(COH)	H
1121	ν(COC) glycosidic, symetric	C
1098	ν(COC) glycosidic, assymetric	C
971	ρ(CH_2_)	C
854	(COC) skeletal mode of α-anomers	P
817	ν(COH) ring	P
478	ν(COC) glycosidic	P
380	δ(CCC) ring	C

**Figure 1:**
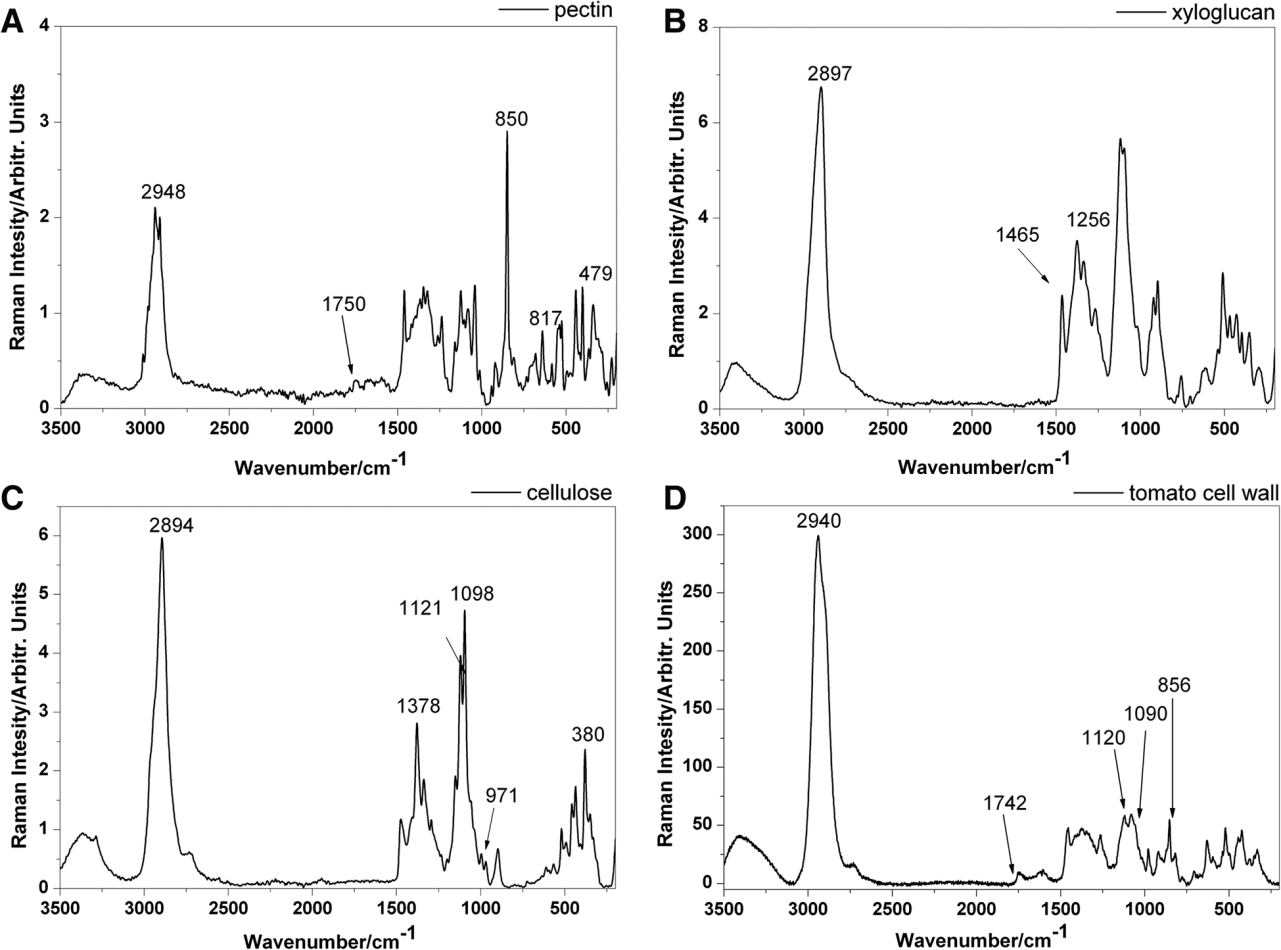
Raman spectra of the pure cell wall components: pectin (A), xyloglucan (B), cellulose (C) and the Raman spectrum of the tomato cell wall (D).

Figure 1 presents the reference Raman spectra of isolated cell wall components (pectins, cellulose and hemicellulose) and the spectrum of material isolated from the tomato cell wall. For the pectins, bands at 2,948, 1,750, 850, 817 and 479 cm^-1^ were assigned (Figure 1A). Meanwhile, for the hemicellulose (xyloglucan), bands at 2,897, 1,459 and 1,256 cm^-1^ (Figure 1B) are shown, while for the cellulose, bands at 2,894, 1,378, 1,121, 1,098, 971, 897 and 380 cm^-1^ (Figure 1C) are presented. In the averaged Raman spectrum of the tomato cell wall material (Figure 1D), many bands characteristic for pure substances were broadened and overlapped and compared to the spectra of the pure polysaccharides (Figures 1A-C). Nevertheless, it was possible to identify and link bands from the tomato’s spectrum to bands characteristic for functional groups of pure polysaccharides. The vibrations assigned for individual cell wall polysaccharides presented in Table 1 were slightly shifted in comparison with the experiment (Figure 1). For example, the bands corresponding to the CH stretching mode are shifted - in the pectins spectrum from 2,952 to 2,948 cm^-1^, and in the cellulose spectrum from 2,897 to 2,894 cm^-1^. Nevertheless, the characteristic bands obtained for isolated cell wall components have been used to visualize the spatial distribution of these polysaccharides from Raman images.

### Single Raman band imaging

**Figure 2:**
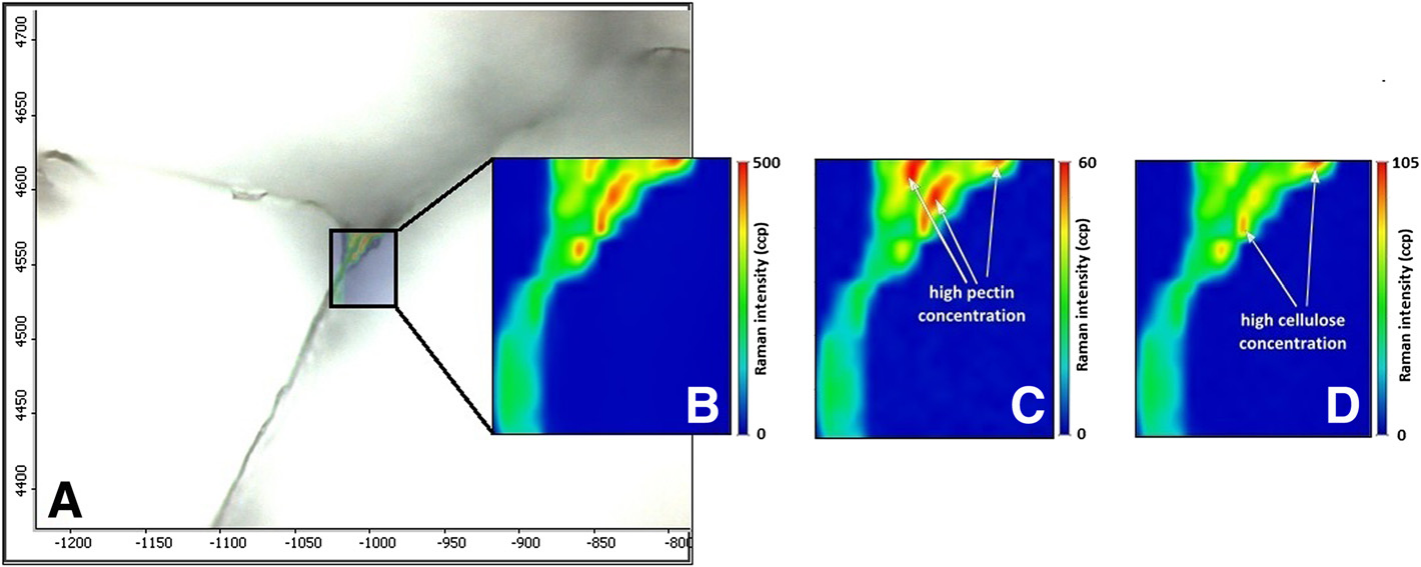
**Images of the tomato cell wall. A)** microscopic image (300 × 400 μm); **B)** Raman image of all primary cell wall polysaccharides at 2, 940 cm^-1^, γ(CH); **C)** Raman image of pectin at 854 cm^-1^, the (COC) skeletal mode of α-anomers; **D)** Raman image of cellulose at 1, 090 cm^-1^, γ(COC) glycosidic.

Figure 2A presents an optical image of size 300 × 400 μm where three cell walls of tomato pericarp tissue are visible. For this experiment, a place on a tomato slice was chosen as an example to analyze the spatial distribution of polysaccharides in tomato cell walls. The recorded map had the dimensions 46 × 54 μm (23 × 27 pixels) and was subjected to a smoothing operation. The map focused on the area of the cell wall corner, since this location is supposed to be rich in pectin. The chemical image (Figure 2B) was obtained by integrating over 2,940 cm^-1^, since this band reflecting the CH-stretching vibrations is characteristic for all primary cell wall components (i.e., cellulose, hemicellulose and pectin) [37].

The chemical maps calculated by integrating over wavenumber ranges corresponding to the strong Raman bands characteristic for individual cell wall polysaccharides are presented in Figures 2C and 2D. The peak at 854 cm^-1^ (Figure 2C) corresponded to the band for the (COC) skeletal mode of α-anomers in pectin [32]. Integration over the 854 cm^-1^ band confirmed higher concentration of pectin inwards of the cell wall. The highest intensity (red color in Figure 2C) was found close to the cell corner of tomato parenchyma. The cell corner and middle lamella are particularly enriched in pectin, especially homogalacturonan. The compounds of this part of the tissue are responsible for cell-cell adhesion [38]. The cellulose distribution is presented at the 1,090 cm^-1^ band (Figure 2D), assigned to the glycosidic bond in cellulose. This band is characteristic for cellulose oriented in parallel to excitation light [32]. Integration over this wavelength displays the largest content of cellulose on the right side of the studied area (red color in Figure 2D). Due to the fact that the sample’s components are homogeneously localized in the studied cell wall, high concentrations of both pectin and cellulose were found in similar locations.

Unfortunately, it was impossible to localize the hemicellulose in this tomato pericarp tissue. The chemical structure of hemicellulose is very similar to cellulose and therefore the bands from the Raman spectrum characteristic for hemicellulose were overlapped by the bands characteristic for cellulose. Moreover, it seems that this group of compounds was distributed homogeneously in the cell wall. The low percentage of hemicellulose in the cell wall material (less than 10%) could be the other reason why the spatial distribution of those components could not be visualized.

### Principal component analysis (PCA)

**Figure 3:**
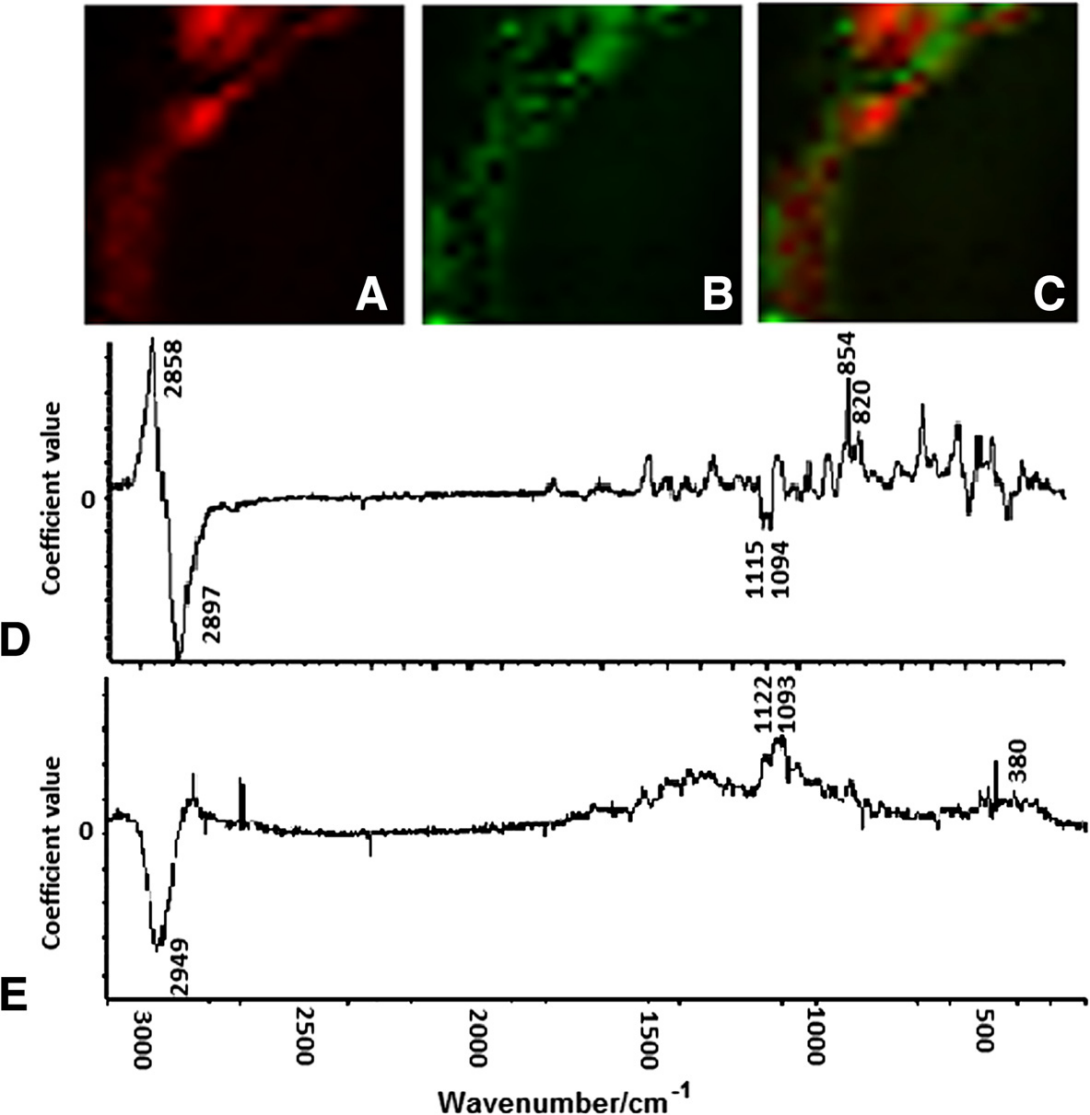
**PCA score images and loading spectra.** Based on comparison with the reference spectra, it was concluded that: **A)** PC2 mainly represents pectins; **B)** PC3 mainly represents cellulose; **C)** PC2 and PC3 are depicted as revealing the distribution of pectins and cellulose; **D)** PC2 loadings; and **E)** PC3 loadings.

Since each polysaccharide is represented by multiple bands in the Raman spectra, single band imaging cannot accurately represent the distribution of polysaccharides in cell walls. The PCA method considers loading from the entire spectra and, therefore, can be used for the imaging of the spatial distribution. For this purpose, loadings for each PC are analyzed to identify which bands had the most influence on the component, such that it is possible to determine which polysaccharide influenced it the most. Under this method, a spatial distribution of the principal components is drawn [39]. The first principal component (PC1) describes the combination of spectral locations with the greatest spectral variance in the map. PC1 typically explains the majority of variability - therefore, PC1 only varies the scores within each group, indicating that it might reflect the standard deviation of the recorded spectra and thus might not be useful for the visualization of the polysaccharides’ distribution [40]. PC1 corresponds to all polysaccharide composites in the sample, without distinguishing between the different compounds.Figure 3 shows the score images and loadings spectra for PC2 and PC3 obtained in this experiment for the studied fragment of the tomato cell walls. Some relationship between the PCs loadings and the pure components (Figures 1A-C) could be established.

PC2 brought information mainly concerning pectin. The large positive loading was observed for the band at 2,958 cm^-1^, assigned to the CH–stretching modes for pectin, while there was a large negative loading at 2,897 cm^-1^ attributed to the CH–stretching modes for cellulose. The second-largest values of the positive loadings was for a pectin marker band at 854 cm^-1^, which might be associated with the skeletal mode of the α-anomers in the pectin molecules. In addition, a band at 820 cm^-1^ (ν(COH) ring) which positively influenced the loading spectrum was observed. Also, bands at 1,115 and 1,094 cm^-1^ which negatively influenced the loadings might correspond with the vibration of the cellulose molecules’ modes, and were present in the PC2 spectrum loadings (Figure 3D).

PC3 predominantly provides information about cellulose. Analysis of PC3’s loading spectrum (Figure 3E) showed that the majority of the bands positively influenced the loadings that were characteristic for cellulose vibration (1,122, 1,093, 380 cm^-1^), and large negative loading was observed for the band at 2,949 cm^-1^ that can be assigned to the CH–stretching modes for pectin.Figure 3C shows the imposed score images for pectin (PC2) and cellulose (PC3). It can be observed that, in some parts, both the PC2 and PC3 scores overlap, but in some places - especially in the middle of the investigated cell wall - the PC2 scores occurred separately. It might be considered that the PC2 scores (red pixels) correspond to the middle lamella – the area between the primary walls, enriched in pectins and responsible for cell-cell adhesion.

Unfortunately, the identification of the hemicellulose’s localization was not possible by PCA analysis. PC4’s loading spectrum (not shown) could not be linked with any polysaccharides from the plant cell wall.

### Multivariate curve resolution (MCR)

**Figure 4:**
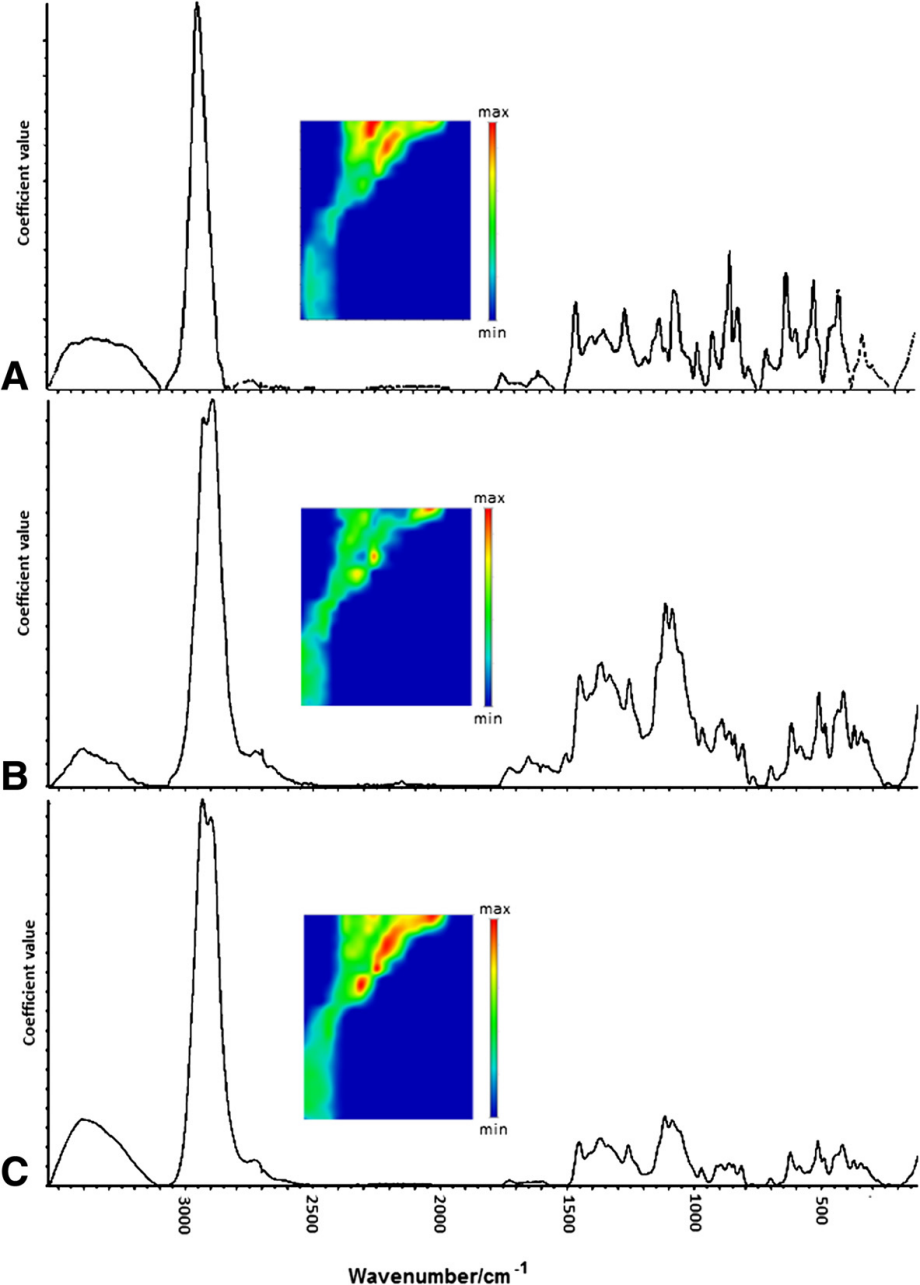
MCR concentration images and the spectra of the sampled pure-components: A) component 1; B) component 2; C) component 3.

The MCR method allows for the recovery of the response profiles of more than one component in unresolved and unknown mixtures, and therefore provides information about the nature and composition of these mixtures. Here, the goal in using the MCR technique was to estimate which pure components are present in the active area map, as well as the locations and concentrations of those 'new’ MCR components. The three main components were extracted and the their Raman spectra and concentration maps could be found (Figure 4).

Based on a comparison of the pure-components’ spectra from the MCR method (Figures 4A-C) and the pure reference spectra of the polysaccharides (Figures 1A-C), the sample constituents could be identified. The similarity between the pectin spectrum (Figure 1A) and the component 1 spectrum (Figure 4A) is noticeable. The spectrum of component 1 contains Raman bands centered at 2,949, 1,751, 855 and 816 cm^-1^ characteristic of pectin polysaccharide (Table 2). Components 2 and 3 could not be clearly distinguished due to the presence in the spectra bands characteristic for cellulose and hemicelluloses. However, it is more likely that component 2 corresponds to cellulose, since its spectrum included more bands characteristic for cellulose molecules’ vibration modes. In the component 2 spectrum (Figure 4B), the bands characteristic for cellulose were centered at 1,378, 1,122, 1,093 and 971 cm^-1^, whereas for hemicellulose they were centered at 2,933 and 1,257 cm^-1^. Additionally, the band at 2,895 cm^-1^ attributed to the CH–stretching modes for cellulose had greater intensity in the component 2 spectrum than in the component 3 spectrum. In the component 3 spectrum (Figure 4C), only bands characteristic of hemicellulose (1,256 cm^-1^) and for both hemicellulose and cellulose (1,459 cm^-1^) were observed.

**Table 2:** **The pure components’ bands (components 1–3) which were detected in the experiments with assignments from the literature**[31-33]**: C – cellulose; P – pectins; H – hemicelluloses**

**Component 1**		**Component 2**		**Component 3**	
**Raman wavenumber [cm**^**-1**^**]**	**Assignment, Origin**	**Raman wavenumber [cm**^**-1**^**]**	**Assignment, Origin**	**Raman wavenumber [cm**^**-1**^**]**	**Assignment, origin**
2949	2948 ν(CH), P	2932	2932 ν(CH), H	2935	2932 ν(CH), H
1751	ν(C = O) ester, P,H	2895	2897 ν(CH), C	2895	2897 ν(CH), C
855	855 (COC)skeletal mode of α-anomers, P	1378	1376 δ(HCC), δ(HCO), δ(HOC), C	1459	1460 δ(CH_2_), δ(COH), C,H
816	817 ν(COH)ring, P	1257	1256 δ(CH) δ(COH), H	1256	1256 δ(CH), δ(COH) ,H
		1122	1121 ν(COC) glycosidic, symetric, C		
		1093	1098 ν(COC) glycosidic, assymetric, C		
		971	ρ(CH_2_), C		
		381	380 δ(CCC)ring, C		

## Conclusions

This study showed that Raman microspectroscopy supported by multivariate image analysis methods is useful in the chemical imaging of polysaccharides’ distributions in the cell wall of the tomato. It was shown that distinguishing between pectin and cellulose was possible from minimally-prepared samples. However, the imaging of hemicellulose’s distribution was not possible due to overlapping characteristic bands with cellulose and pectins.

## Methods

**Figure 5:**
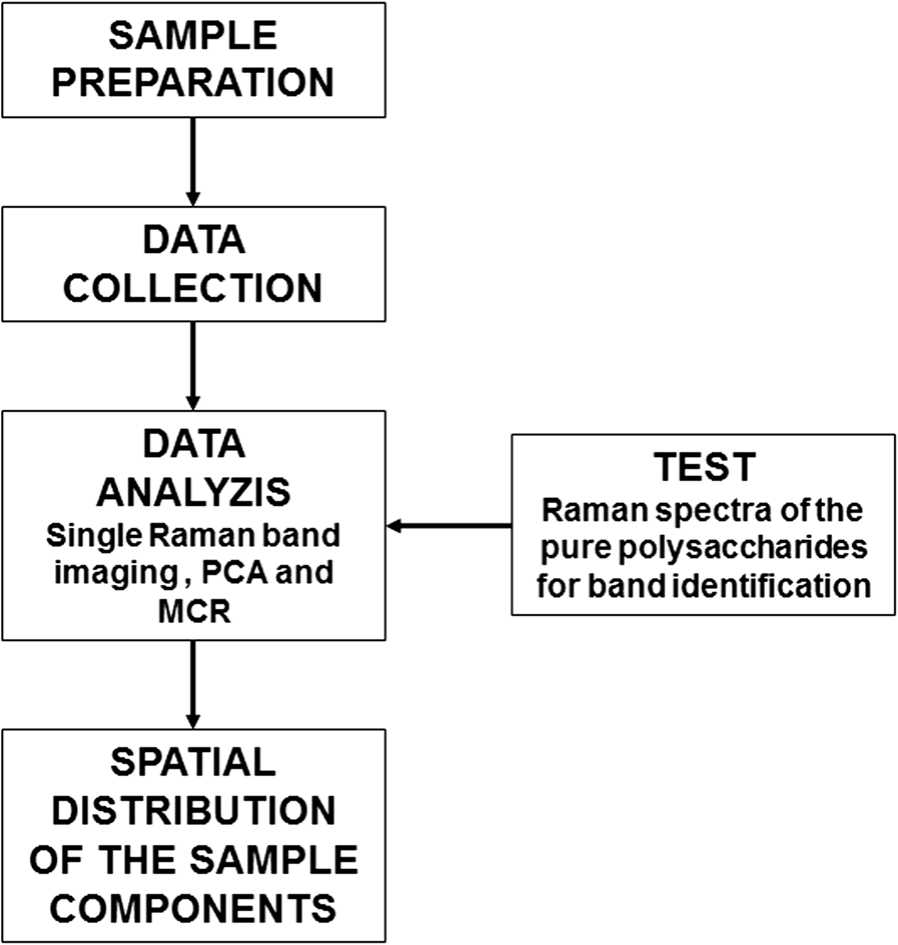
Scheme of the experimental procedure.

The main steps of the experimental procedure are presented on the scheme below (Figure 5). Each step is described in detail in what follows.

### Sample preparation for Raman imaging

The fully ripe fruit of tomato (*Lycopersicon esculentum* Mill. Cv Conqueror) was used in the experiment. In preliminary experiments, a method for sample preparation was developed to study fruit tissue with Raman microspectroscopy. Prior to cutting, tomato parenchyma tissue was frozen and then lyophilized. The lyophilized tissue was cut using a vibratome (Leica VT 1000S) into slices of a thickness of 130 μm. The cut slices were then soaked in acetone in order to remove pigments from the tomato tissue. The samples were placed on a microscope glass slide which, beforehand, was covered with aluminum foil to avoid interference from the glass Raman spectrum on the sample map.

### Raman spectra of the reference materials

Additionally, to obtain exemplary Raman spectra of individual polysaccharides, commercially available pectins, hemicelluloses and cellulose were studied. High methylated (degree of methylation 80%) pectins were purchased from Herbstreit and Fox (Neuenbürg, Germany), microcrystalline cellulose powder (ca. ~ 20 μm) from Sigma Aldrich and xyloglucan (tamarind, purity >95%) from Megazyme (Bray, Ireland). All the chemicals were used without further purification. Also, the Raman spectrum from the tomato cell wall was collected for comparison. Characteristic bands of the individual polysaccharides from the reference materials were used for the calibration of the Raman imaging method.

### Raman microscope

The imaging system used in this study was a DXR Raman Microscope (Thermo Scientific, Waltham, USA), equipped with a diode-pumped, solid state (DPSS) green laser (λ = 532 nm) with a maximum power of 10.0 mW, a diffraction grating of 900 lines per mm and a pinhole confocal aperture of 25 μm. The Raman light was detected with an air-cooled CCD detector with a spectral resolution of 4 cm^-1^. The 20x/0,40NA objective was used.

The map was recorded with a spatial resolution of 2 μm in both directions, x, y. The vertical z displacement was fixed. The integration time (8 s) was fixed for each scan. A single spectrum at each point was recorded within the range of 3,500–150 cm^-1^ of Raman shift for an average of 12 scans. Each pixel corresponds to one average spectrum. The spectra were not normalized.

Also, the Raman spectra of the reference materials were collected on a DXR Raman Microscope (Thermo Scientific, Waltham, MA, USA), with a green laser (λ = 532 nm) and a maximum power of 10.0 mW. The spectra were recorded within the range of 3,500–150 cm^-1^.

### Visualization methods

The Omnic Atlμs program (Thermo Scientific, Omnic 8.1, USA) was used in collecting the Raman spectra and performing the data analysis in order to obtain chemical images. Moreover, PCA and MCR were performed using the Omnic program.

Single Raman band imaging allows the generation of two–dimensional images based on the integral of the different Raman bands that are characteristic for sample components. It is used for the preliminary analysis and initial identification and localization of the biopolymers in the sample.

PCA is mathematical technique used for reducing the dimensionality of data from hundreds of spectral data points into a few orthogonal PCs. Each PC explains a part of the total information contained in the original data but not always corresponding to one specific chemical component (especially when several pure components’ spectra are overlapping) [41]. The first basis spectrum - or principal component - accounts for the maximum variance in the data if the data is mean-centered prior to analysis. The second basis spectrum accounts for the next most variance, and so on. These spectra bases are created such that they are orthogonal to each other and, therefore, contain no overlapping spectral information. These principal components are fitted to the imaging data set and are used to create a two-dimensional image, which will provide a map of how the spectral features represented by the principal components are distributed in the sample. This map can be correlated with the Raman spectra of known chemicals [42].

Another exploratory analysis of the chemical data (especially the spectroscopic and chromatographic data) is given by MCR [43]. This technique allows for the estimation of which pure components (and, therefore, chemical species) are present in the active area map and shows the locations of those components. The aim of MCR is to mathematically decompose an instrumental response for a mixture into the pure contributions of each component involved in the system studied [44]. MCR maximizes the explained variance in the data - as PCA would - while also delivers physical or chemical information about the system rather than the mathematical or statistical constraints as for PCA. The profiles of the pure-components are given a chemical meaning and can be straightforwardly interpreted as concentration profiles and spectra [43]. In the results of the analysis, the pure-components’ spectra are obtained. Another important feature of MCR is that although the method itself does not require prior knowledge concerning the sample, additional information can always be incorporated to facilitate the analysis when available. For example, the reference spectra of known existing components can be used as an initial estimate. However, the MCR method requires an estimation of the number of pure components in the system [41].

Single Raman band imaging allows the generation of images of the studied component but only based on the one Raman band from the spectrum. Therefore it is used for the preliminary analysis. Whereas PCA and MCR methods relate to the data from the entire spectrum or from the range selected from the spectrum. Those methods deliver more details and are much more reliable than imaging based on one band (even characteristic for studied component).

For PCA, two regions of the spectra were selected (3,500–2,500 and 1,800–200 cm^-1^) and four principal components were analyzed. MCR analysis was performed using the whole spectra and three pure components were provided.

The graphical presentation of the individual spectra was prepared on the OriginPro program (Origin Lab v8.5 Pro, Northampton, USA).

## Abbreviations

MCR: Multivariate curve resolution; PCA: Principal component analysis.

## Competing interests

The authors declare that they have no competing interests.

## Authors’contributions

MC and MS performed the experiment. MC wrote the manuscript. MS and AZ participated in the preparation of the manuscript. MS and AZ supervised the experiment and preparation of the manuscript. All authors read and approved the final manuscript.
